# American Public Health Association

**Published:** 1896-10

**Authors:** 


### Society Proceedings.

### AMERICAN PUBLIC HEALTH ASSOCIATION.

*Proceedings of the Twenty-fourth Annual Meeting, held in Buffalo, N. Y., September 15–18, 1896.*

FIRST DAY—MORNING SESSION.

THE association assembled in Ellicott Square and its deliberations were presided over by Dr. EDUARDO LICÉAGA, of Mexico, president of the Superior Board of Health of that city.

Dr. STEPHEN SMITH, of New York, the first president of the association, was introduced and made a few remarks with reference to the progress the association has made from its beginning.

Dr. ERNEST WENDE, commissioner of health, of Buffalo, cordially welcomed the association in behalf of the local committee of arrangements.

Report of the committee on car sanitation: This was read by Dr. C. O. PROBST, in the absence of Dr. G. P. Cohn, of Concord, N. H., chairman. The report states that anyone who takes an interest in car sanitation will soon become convinced that there is something lacking in the manner in which cars are cleansed and kept in condition for the traveling public. In caring for these conditions, ignorant and untrained help may and do destroy a great deal that should be cared for, and thus the expenses of this department are far beyond what is actually necessary.

Observations on the cleaning of railroad passenger cars, by Dr. DOMINGO ORVAÑANOS, of the City of Mexico: To afford any security against contagion or infection from railroad cars, it is necessary that the cleansing operations shall be carried out several times a day. To attain these objects, the author thinks passenger cars ought to be constructed in a very different manner than they are at present. The bed clothing, including the blankets and curtains, should be changed daily, as well as the mattresses.

Possibilities of contagion from venereal diseases in railway cars: This paper was read by Dr. TOMÁS NORIEGA, of the State of Chiapas, Mexico, in which he cited the case of a married man, thirty years of age, who arose from his berth, and as was his custom, washed his face in the lavatory of a Pullman car. Two days thereafter he felt the first symptoms of purulent ophthalmia, for which he consulted a physician. The patient was treated energetically, but in spite of all efforts the right eye was lost. Other similar cases were reported.

Dr. FREDERICK MONTIZAMBERT, of Montreal, general superintendent of the quarantines of the Dominion of Canada, presented the report of the committee on steamboat and steamship sanitation.

AFTERNOON SESSION.

A paper on the Infectiousness of milk was read by Dr. JAMES KENNEDY, of Des Moines, Iowa. Cow's milk alone was considered, since no other kind of milk is used by many infants and adults, and since it is the almost universal, and, under proper conditions, the best substitute for human milk in the feeding of children. In Berlin, in giving the certificates of death of children under one year, the fact must be stated as to whether the child was fed from the breast or brought up artificially. In 10,000 deaths thus reported it was found that two-thirds, or 7,646, were artificially fed. The author emphasised the importance of a sanitary inspection in addition to, if not to the exclusion of, the mere commercial examination.

Report of the committee on animal diseases and animal food: This was read by the chairman, Dr. D. E. SALMON, of Washington, D. C. Animal diseases are now more intelligently managed by sanitary officers than ever before, and the meat inspection service has been steadily extended and perfected. Outbreaks of anthrax among the domesticated animals were apparently becoming more frequent. The contagion once introduced into a pasture remains indefinitely. A disease so fatal to man and beast should be promptly repressed whenever it makes its appearance and precautions observed to prevent infection of new territory. The report then dwells upon the widespread prevalence of tuberculosis among dairy cattle. Measures for the eradication of this disease have been undertaken by a few states and by a considerable number of cities.

Dr. FRANCISCO DE P. BERNALDEZ, of Mexico, contributed a paper on The study of the pathogeny, etiology and prophylaxis of typhus. This disease arises from a microbe not as yet discovered. Throughout all the districts which are called the hot country in the Mexican Republic the infection of typhus does not exist, whilst in temperate climates, at a higher elevation, it predominates in endemic form.

Report of the committee on nomenclature and forms of statistics, by Dr. SAMUEL W. ABBOTT, of Wakefield, Mass., chairman: The report dealt with the need of a uniform system of classification and nomenclature. Before advising the general acceptance of any one system for general use, the committee recommended that the association collect and compare the systems now in use and employed by the different national, state and municipal authorities in this country, in order that these may also be compared with the systems in use in other countries, so that a general system can be recommended for adoption throughout the states and countries within the bounds of the association.

Dr. EDUARDO LICÉAGA, the president, read a paper on The nomenclature of diseases and forms of statistics. The board of health of the City of Mexico had, from the year 1879 up to the year 1887, classified the diseases resulting in death in a certain number of groups. From the year 1888, he, as president of the board, proposed the adoption of the provisional nomenclature adopted by the Royal College of Surgeons of London. This nomenclature was adopted because it was the one then followed by almost all the English-speaking nations, and in order that tables of mortality might be compared with those of such nations.

On the need of uniformity in the meaning of the term stillborn, by Dr. JESUS E. MONJARÁS, of San Luis, Potosi, Mexico. The laws of different countries were cited by the author, after which he proposed the following: (1) That there shall be included under the term stillborn all children of more than six months of intrauterine life that are born dead. (2) That there be added to the nomenclature of the causes of death the term that shall represent all children that die within seventy-two hours after birth without known cause, and that they be designated by the term "died at birth without known cause." (3) That the committee on nomenclature of diseases and forms of statistics be authorised to recommend this modification of the existing nomenclature in all the countries of the American continent, and (4) that these modifications once adopted in said continent, the same would doubtless be accepted in Europe and elsewhere.

Dengue: A paper on this subject was read by Dr. HENRY D. HORLBECK, of Charleston, S. C. The disease was defined, after which the author said the object of the paper was to put on record a brief account of a widespread outbreak of this malady, which occurred in Charleston in 1895. Commencing in July and lasting until November, it is estimated that 50,000 of the inhabitants were afflicted with the disease. Men and women, seventy years of age, and infants, had it, and yet the malady was not prevalent a few miles distant. Notwithstanding the suddenness of the onset and severity of the attack, death is rare.

Municipal responsibility for healthy school houses: Mrs. ELLEN H. RICHARDS, of Boston, contributed a paper on this subject. Local agitation of this question might do some good, but to the author it seemed as if the time had come for some concerted action compelling city authorities to keep school houses in good condition. A most efficient way would be to bring to bear the power of the law and to insist that such buildings as are flagrant violations of the law shall be closed, as private buildings would be.

EVENING SESSION.

Addresses were delivered by the MAYOR of Buffalo and the Rev. THOMAS SLICER, both of whom spoke of the benefits of sanitation.

The president, Dr. LICÉAGA, then delivered his address. He first thanked the members for the distinguished honor conferred upon him, after which he said that the preservation of health, the prolongation of life and the physical improvement of the human race were the ideal principles that ought to be kept in view.

Coming to the question of epidemics, President LICÉAGA stated that they can be suppressed at their inception by isolating the first patients and disinfecting the objects which they have contaminated, whether these objects be the clothes they have used, the furniture found in their respective rooms or the rooms in which they were kept during the disease. Isolation in cases of diphtheria must be absolute and complete.

A proposition which demanded special study was the technique of disinfection.

Lastly, the speaker cited examples to show the advisability of organising a committee to study the periods during which each contagious disease is transmissible, and the time during which every patient who has suffered from such disease is dangerous to the community.

### SECOND DAY—MORNING SESSION.

Report of the committee on the disposal of garbage and refuse: This was presented by Mr. Rudolph Hering, C. E., of New York City, chairman, and was followed by a paper entitled Disposal of the garbage and waste of the household, by Col. W. F. Morse, of the same city. In considering the matter of the final disposition of garbage, the author said that no record of methods could be complete unless those means were considered by which the waste of the family was destroyed in the home where it was produced. An apparatus in the form of a carboniser for the disposal of garbage was described.

Dr. N. E. Wordin, of Bridgeport, Conn., read a paper entitled A plea for the domestic disposal of garbage. Fire is the best destroyer. It leaves no filth and no germs behind. The different methods of disposing of garbage were tabulated as follows: (1) The most wasteful—sea disposal. (2) The most offensive—hog feeding or fertilisation. (3) The most economical to operate—reduction. (4) The most sanitary and complete—cremation. Reduction and cremation were the only methods worthy of consideration for any city.

Dr. Wm. S. Tremaine, of Buffalo, explained the results of practical experiments with one of the garbage crematories in Buffalo. This crematory successfully disposes of garbage and excrement without any odor.

Report of the committee on transportation and disposal of the dead, by the chairman, Dr. Charles O. Probst, of Columbus, O.: The committee is of the opinion that it is quite possible to so prepare a body, dead of infectious diseases, with promptitude and but little expense as to make it transportable without any danger of transmitting infection, and it is the duty of the association to develop the simplest methods by which this desirable end could be obtained, in order that the sentiment of respect for the dead might be maintained without any danger to the living. If, however, all dead bodies are to be allowed transportation, it would be necessary to provide that the preparation of bodies, whose death resulted from contagious disease, shall in each instance be under the direct supervision of the health authorities.

The quick or the dead: Dr. BENJAMIN LEE, of Philadelphia, read a paper with this caption. Health authorities should be very slow in relaxing any of the precautions and restrictions at present in force attending the transportion of those dead of contagious diseases. He thinks the true solution to the question of transportation is to be found in the cremation of all bodies dead of contagious diseases.

On measures for the prevention of blindness, by Dr. AUGUSTIN CHACON, of the City of Mexico: Statistics, cited by the author, prove that a great deal more than half of the cases of blindness might very probably have been avoided, if proper measures had been taken in time. The two diseases of the eyes which cause the loss of sight in the largest number of patients were atrophy of the optic nerve and purulent ophthalmia. These two diseases were considered at length. Special attention ought also to be given to hygiene of the sight in schools.

Dr. ALBERTO G. NORIEGA, of Mexico, read a paper on Miasmatic fevers in the State of Sonora. The author spoke of the origin, treatment and some of the peculiarities of the symptomatic characteristics of fevers from miasmatic origin in this state. He proposed the following prophylactic measures: (1) The planting of thick woods around the township, with the idea of suppressing the paludic miasma where the trees grow. (2) The houses ought to be built on the highest places, in order to keep them as far as possible out of the reach of the gases from the pools and marshes. (3) The front of the houses must not face the direction of the dominant wind, and the houses themselves ought not to be in the way of the wind coming from the pools. (4) To avoid the watering of the floors, in order to maintain the interior of the houses as dry as possible. (5) The workmen in the fields must not commence their work until the sun is way up and they must retire from the field before the sun sets.

Summary of sanitary legislation in the State of Mexico. This paper was read by Dr. M. ALVAREZ, of Mexico. The author said that the philosophy of sanitary legislation rested on three bases: (1) Those which attempt to endow the individual with good health. (2) Those which take precautions against diseases of all kinds. (3) Those which require the partial sacrifice of individual liberty in favor of the general community. The author then entered exhaustively into drinking waters, vaccination and vaccination laws, paying particular attention to the obligatory vaccination law of Mexico.

AFTERNOON SESSION.

Dr. A. WALTER SUITER, of Herkimer, N. Y., read a paper entitled Obiter dicta, concerning sanitary organisation. He said a system of health administration without effective organisation was like a ship without a rudder, subject to the mercy of every pestilential storm. Dr. Suiter made a strong plea for an arrangement so systematised that sanitary direction may be administered in the most practical and advantageous manner without conflict of authority. The public should be educated to a point of proper appreciation of the importance of the service required.

Dr. U. O. B. WINGATE, of Milwaukee, Wis., read a paper entitled Some thoughts relative to sanitary legislation. The author believes that laws pertaining to sanitation should differ very materially from other laws, inasmuch as they voice a scientific fact, and if applicable in one locality they should be also applicable in all localities. Attention was directed to the great need of a system of statistics, not only pertaining to births and deaths, but to sickness or the prevalence, especially of contagious and preventable diseases. A strong plea was made for a department of public health at Washington.

In a paper entitled The sanitary administration of unincorporated districts, Dr. HENRY MITCHELL, of Trenton, N. J., presented the following propositions: (1) By law provide that in each township or other local political division outside of municipalities, the sanitary authority should be exercised by one official. (2) The local health officers should be selected under civil service rules and their term of office should be five years. (3) The examination of applicants for the office of township health officer should be conducted by the state board of health. (4) The appointment of the health officer in each township should be made by the governing body of the district from an eligible list to be furnished by the state board of health. (5) No health officer should be removed except for cause, and vacancies should be filled for the unexpired term in the manner provided for original appointments. (6) Local health officers should be required to conduct all of their official operations in accordance with rules and regulations approved by the state board of health, and they should also make weekly reports of their doings to said board and annually to the local governing body. (7) The local health officer should be paid for his services by the local governing body. (8) All suits for the violation of any local sanitary rule, regulation or ordinance, should be brought at the instance of the local health officers, and they should be prosecuted by the district attorney or prosecutor for the county, but no such suit should be begun until the necessity for its being instituted has first been agreed to by the state board of health.

Report of the international committee on the prevention of the spread of yellow fever, by the chairman, Dr. Felix Formento, of New Orleans: Regarding this disease, the recommendations of the committee we give herewith: (1) Extreme measures of local sanitation in yellow fever foci; modification of the soil, improvement of harbors, and the like, by all means known to sanitary engineering. (2) Putting in perfect sanitary condition all home seaports and towns most exposed to infection. (3) A rigid and efficient system of quarantine against the introduction of the disease. (4) Abolishing forever the abominable system of interment and disinterment practised in Spanish-American countries. (5) Wherever practicable, yellow fever hospitals should be established beyond or above yellow fever foci; when this cannot be done, these hospitals should be established at a distance from centers of population in a desirable locality and perfectly isolated. (6) Compulsory cremation of all bodies of persons who have died of that disease and incineration of all infected material.

The study of yellow fever from a medico-geographical point of view, by the president, Dr. Licéaga: This was the fourth paper he had presented on this subject, and his object was to enable the association to realise the true situation of the Mexican republic as regards yellow fever. With the aid of facts he dissipated the erroneous idea which for so many years had existed, that it was a country in which this disease was always found throughout the entire extent of its territory.

Dr. G. MENDIZABAL, of Orizaba, Mexico, followed with a paper entitled A contribution to the study of yellow fever in relation to epidemics in Cordova. The author presented a resumé of the number, intensity, duration and mortality of each of the epidemics of yellow fever which had desolated, during three centuries, the above-mentioned city. This city, besides its climate and soil, its constant humidity, its proximity to Vera Cruz, and many other causes which favor the propagation of the modific germs, has a great scarcity of potable water of the requisite purity. It is the duty of the municipal authorities to improve the hygienic conditions of the people of this city, to provide them with potable water, to make the soil sterile to the germs of the disease, and thus forever close the doors against this desolating plague.

Dr. JOHN L. LEAL, of Paterson, N. J., read a paper on Isolation hospitals, in which he spoke of their utility in the restriction of preventable diseases, and illustrates his remarks by views and plans of the Paterson Isolation Hospital.

THIRD DAY—MORNING SESSION.

Major CHARLES SMART, Surgeon of the U. S. A., Washington, D. C., chairman, read the report of the committee on the pollution of water supplies, in which he referred to the bacteriological convention held in New York City, and the work accomplished by it, and says that when the standard methods of this convention are in the hands of the bacteriologists of this country, the committee will then be in a condition to define its lines of action for effecting an organisation for coöperative work, as suggested at Montreal.

Dr. PETER H. BRYCE, of Toronto, chairman, presented the report of the committee on river conservancy boards of supervision. The committee was not as yet prepared with such data regarding individual cases of pollution to present practical suggestions with reference to such a board for any particular stream, but desired, by laboring in conjunction with the committee on pollution of streams and various engineering associations, to collect material which might give to the committee's report, in another year, some practical value.

Dr. CHARLES N. HEWITT, of Red Wing, Minn., as chairman, presented the report of the committee on protective inoculations in infectious diseases.

The serum diagnosis test for typhoid fever, by Dr. W. JOHNSTON, of Montreal : The author demonstrated a modification of Vidal's method of serum diagnosis in this disease. He considers the test very reliable from a diagnostic point of view and thinks it will prove of considerable value for public health work, and that it will have a tendency for physicians to more frequently and promptly report their cases.

Prophylaxis of typhoid fever was the title of a paper by Dr. JOHN E. WOODBRIDGE, of Cleveland, O. Typhoid fever was characterised as a water-borne disease, and every attack was considered the child of a previous one and was *prima facie* evidence that the victim had eaten or drank unsterilised human excrement or some of the products thereof. The Government of the United States will not have discharged its whole duty to the people, will not have attained the zenith of its greatness, until through a Department of Health, aided by wise legislation, it has taken every possible precaution to protect the health and foster the highest physical development of her citizens, by guarding well the purity of the air they breathe, the food they eat and the water they drink.

Dr. F. C. ROBINSON, of Brunswick, Maine, read a paper in which he spoke of the practical use of formic aldehyde as a disinfectant.

Dr. E. A. DE SCHWEINITZ, of Washington, D. C., demonstrated and exhibited a convenient lamp for generating formaldehyde gas; while Dr. J. J. KINYOUN, of Washington, D. C., followed with a preliminary note on the use of formaldehyde for room and car disinfection. His results so far obtained from its use were very gratifying. Dr. Kinyoun also exhibited and described an apparatus of his own design for generating this gas.

Two papers were then read, one on The prophylaxis of paludism by Dr. A. R. ERDOZAIN, of Mexico, and the other on Paludism in the State of Morelos and its prophylaxis by sanitary measures, by Dr. A. GAVINO, of Mexico.

Dr. JUAN MULDSON, of Mexico, read a paper on Public health in Tabasco. He presented the following conclusions : (1) The sanitary condition of Tabasco in general is good. (2) Paludism is the principal disease, but it is satisfactorily treated. (3) Yellow fever is not endemic ; it occurs in isolated cases, being generally imported and not finding a good soil for its propagation. (4) Isolation and other hygienic measures have successfully prevented propagation of the disease. (5) Natives are not as easily attacked by yellow fever as foreigners are, and this immunity reaches people accustomed to the climate who have lived there for many years. (6) The climatological conditions notably modify the clinical history of certain diseases, among which forcibly calling our attention is the benign course of septicemia.

AFTERNOON SESSION.

Dr. J. J. KINYOUN, of Washington, D. C., chairman, presented the report of the committee on the cause and prevention of diphtheria. The committee recommends the following:

1. That there should be uniform rules and regulations adopted by all the states and provinces for the prevention and control of diphtheria. The several governments should assume the responsibility and act in unison in preventing the spread of the disease from one country to another and assume authority over inter-provincial and inter-state communication.

2. That it should be the duty of the health authorities to provide facilities for determining the diagnosis in all suspected cases by the establishment of inexpensive laboratories for each health jurisdiction. To agree upon a system and means of transmission of material for diagnosis through the mails.

3. Compulsory notification of all suspected cases and the abolition of the terms croup and membranous croup, unless diphtheria has been excluded by culture and microscopic examination.

4. Compulsory isolation of all cases, domiciliary or in hospital, until the recovered cases show the absence of the diphtheria bacillus.

5. That the medical inspection of schools should be inaugurated under the direction and supervision of the health authorities, by making daily inspections in the larger cities of all school children for the detection of infectious diseases. (The plan advocated by Dr. S. H. Durgin, of Boston, at the last meeting of the association was highly commended.)

6. School buildings, books, and the like, should be subjected to a reliable method of disinfection at least once per month, and oftener if suspected of being infected.

7. The early treatment of those ill with diphtheria with antitoxin, the administration of preventive doses to those who have been exposed to infection and have the bacilli in their throats.

8. Prompt and effective methods of disinfection of infected articles and apartments, to be carried out under the supervision of the health authorities.

Dr. Marquez, of Mexico, contributed a paper on Diphtheria in Chihuahua. He said that diphtheria was one of the infecto-contagious diseases which was most observed in Chihuahua, and in such a degree that it sometimes caused a panic among families. The author lays down twenty-four rules to be carried out to prevent or avoid the spread of the disease.

Bacteriological diagnosis as governing the admission and discharge of patients in diphtheria hospitals, by Dr. E. B. Shuttleworth, of Toronto. The isolation hospital at Toronto was established in 1891, and up to June 30th last there were admitted 1,690 patients, said to be suffering from the disease. Diagnosis by bacteriological methods was begun in February, 1895, and since July of that year the discharge of patients had also been governed by this means of investigation. The statistics for this period covered 565 cases, and when compared with those for the preceding time afforded an opportunity for ascertaining the practical value of bacteriology when applied to the purposes indicated.

Dr. Charles N. Hewitt, of Minnesota, presented the report of the committee on causes and prevention of infant mortality.

Dr. S. Garciadiego, of Mexico, followed with a contribution on The mortality of children, its causes and means of diminishing it. The speaker classified the causes of mortality of children under three heads—crime, carelessness and ignorance. The author believes that the mortality among children can be diminished by the institution of lying-in hospitals or obstetrical departments, by which means it has been proved that infanticide nearly disappears. By establishing orphanages and homes for foundlings under the care of the government, and also under the protection of the public beneficiary, and under the care of religious people and especially the parishes. In each of these asylums a limited number of children should be allowed in order to be properly cared for and attended to. For the feeding of infants in these institutions the mother's milk should be replaced by that of the goat or other nearly as proper as the former, using the utmost care in the cleaning of bottles. Mothers who abandon their children should be severely punished.

EVENING SESSION.

Dr. Felix Formento, of New Orleans, presented the report of the committee on the use of alcoholic drinks, which was substantially that presented last year at the Denver meeting.

Dr. Albert L. Gihon, of New York, read a paper on The bicycle in its sanitary aspect. The author criticised the posture and saddles used by riders of the bicycle. After presenting arguments for and against the bicycle, he ventured the prediction that a light three or four wheeled vehicle, impelled by some easily managed motor, inexpensive enough to be generally available, would be the means of progression for pleasure purposes in the future, covering long distances without fatigue, permitting sight-seeing and outdoor exposure without labor, and adding the charm of companionship and participated enjoyment, while the rational instrument of exercise for exercise's sake alone would ever be a pair of sturdy human legs.

Dr. H. L. Chase, of Brookline, Mass., read a paper on Public bathing establishments, and gave a description of the new public bath in Brookline; while Dr. W. H. Tolman, of New York, gave an illustrated lecture on Public baths.

Dr. Carlos Santa-Maria, of Durango, Mexico, read a paper on The part that public instruction should play in the way of precaution against contagious diseases. It was a plea for the general teaching of the elements of hygiene in the public schools.

FOURTH DAY—MORNING SESSION.

At this session the following papers were read:

Report of committee on relation of forestry to public health, by Prof. R. C. Kedzie, Lansing, Mich.

Report of committee on transportation of diseased tissues by mail, by Dr. Henry Mitchell, of Trenton, N. J.

On statistics of vaccination and mortality from small-pox in the City of Mexico, 1887–1895, by Dr. Jose Ramirez, of Mexico.

Dr. A. N. Bell, of Brooklyn, N. Y., read a paper on Drunkenness, which he considered as a vice, and said that it should be so treated.

Dr. Wm. C. Krauss, of Buffalo, N. Y., read a paper on The relation of noises to public health, published on page 184 of the Journal.

Dr. F. C. Valentine, of New York City, read a paper on The protection of the innocent from gonorrhea. He said that if justification were needed for the discussion of this matter it could be found in the statistics of the German Empire for 1894. These show that of the women who died of uterine or ovarian diseases, 80 per cent. were killed by gonorrhea. They further show that of children hopelessly blind, 80 per cent. went into a life of darkness from gonorrhea. Gonorrheal patients should be educated in incontrovertible facts, the physician ever choosing terms within the range of their intelligence.

Several other papers on the program were read, some of them by title.

The following officers were elected for the ensuing year: President, Dr. H. B. Horlbeck, of Charleston, S. C.; first vice-president, Dr. Peter H. Bryce, of Toronto; second vice-president, Dr. Ernest Wende, of Buffalo; treasurer, Dr. Henry D. Holton, of Brattleboro, Vt.; secretary, Dr. Irving A. Watson, of Concord, N. H.

Place of meeting, 1897, Philadelphia.

